# Parecoxib Reduces Systemic Inflammation and Acute Lung Injury in Burned Animals with Delayed Fluid Resuscitation

**DOI:** 10.1155/2014/972645

**Published:** 2014-01-21

**Authors:** Si Jack Chong, Yong Chiat Wong, Jian Wu, Mui Hong Tan, Jia Lu, Shabbir M. Moochhala

**Affiliations:** ^1^Naval Medical Service, Singapore Armed Forces, Singapore; ^2^Combat Protection and Performance Lab, Defence Medical & Environmental Research Institute, DSO National Laboratories (Kent Ridge), 27 Medical Drive No. 12-01, Singapore 117510

## Abstract

Burn injuries result in the release of proinflammatory mediators causing both local and systemic inflammation. Multiple organ dysfunctions secondary to systemic inflammation after severe burn contribute to adverse outcome, with the lungs being the first organ to fail. In this study, we evaluate the anti-inflammatory effects of Parecoxib, a parenteral COX-2 inhibitor, in a delayed fluid resuscitation burned rat model. Anaesthetized Sprague Dawley rats were inflicted with 45% total body surface area full-thickness scald burns and subsequently subjected to delayed resuscitation with Hartmann's solution. Parecoxib (0.1, 1.0, and 10 mg/kg) was delivered intramuscularly 20 min after injury followed by 12 h interval and the rats were sacrificed at 6 h, 24 h, and 48 h. Burn rats developed elevated blood cytokines, transaminase, creatinine, and increased lung MPO levels. Animals treated with 1 mg/kg Parecoxib showed significantly reduced plasma level of CINC-1, IL-6, PGEM, and lung MPO. Treatment of 1 mg/kg Parecoxib is shown to mitigate systemic and lung inflammation without significantly affecting other organs. At present, no specific therapeutic agent is available to attenuate the systemic inflammatory response secondary to burn injury. The results suggest that Parecoxib may have the potential to be used both as an analgesic and ameliorate the effects of lung injury following burn.

## 1. Introduction

Burn injury results in increased microvascular permeability leading to edema formation due to the loss in plasma fluid to the interstitial space [1]. Timely and early resuscitation of burn shock is effective in restoring normal tissue perfusion. However, severe burn victims are in pain, uncooperative, and unable to seek help that may delay evacuation. When resuscitation is delayed, recovery can be complicated by multiple end-organ failure due to systemic inflammation after severe burn and systemic inflammatory response syndrome (SIRS) has remained the primary cause of death in critically injured patients, with acute respiratory distress syndrome (ARDS) as the leading cause [2, 3]. An early management of burn injury is essential for the good prognosis of burn patient. It has been reported that early control of inflammation after burn improves pulmonary function and survival [4, 5]. Consistent in these findings, Cyclooxygenase-2 (COX-2) has been shown to mediate inflammatory process through prostaglandins release after burn injury and inhibition of COX-2 attenuated proinflammatory cytokines, chemokines, and acute lung injury in burn-injured mice [6–8]. Parecoxib, a nonsteriodial anti-inflammatory drug (NSAID) indicated for postoperative pain, selectively blocks off the action of COX-2 enzyme has been of interest on early burn management [9]. Recently, it was shown that burn injury induces COX-2 expression in central nervous system [10] and Parecoxib has been shown to have neuroprotective properties [11], although anti-inflammatory drugs for early burn management have been proposed but hitherto poorly studied and the effects of NSAID on SIRS after burn injury remain largely unknown. If effective, Parecoxib can be used both as an analgesic and ameliorate the effects of SIRS and ARDS in early burn management. On the basis that there are no reports on the effects of anti-inflammatory agent in the early management of burn injury with delayed resuscitation, the development of a rat model that consistently demonstrates burn-induced SIRS/ARDS and survives for 48 hours sufficient for the evaluation of acute burn management modalities was conducted. The treatment efficacy of Parecoxib in animals following acute burns injury was evaluated in the present study.

## 2. Material and Methods

The experimental procedures were approved by DSO National Laboratories Institutional Animal Care and Use Committee.

### 2.1. Burn Injury Rat Model

Adult male Sprague Dawley rats (325–350 g) were acclimatised for one week before experiments. Animals were anesthetized with 100 mg/kg Ketamine and 7.5 mg/kg Diazepam, intraperitoneally (i.p.). The dorsal and ventral surfaces of the animals were shaved with clippers. The rats were placed into a customised mould that exposed 45% of the total body surface area (TBSA) calculated based on Meeh's formula [12]. Previous studies have shown that severe burn of more than 40% TBSA inflicted ARDS and SIRS in humans [2, 3, 13] and animals [14–16]. The mould was placed in 98°C to 100°C water, scalding the back for 10 s and the abdomen for 4 s. Forty mL/kg of Hartmann's solution (131 mmol/L Na^+^, 5 mmol/L K^+^, 2 mmol/L Ca^2+^, 111 mmol/L Cl^−^, and 29 mmol/L HCO_3_^2−^, B. Braun Melsungen AG, Melsungen, Germany) was administered i.p. half at 1 h after burn and the remaining half at 8 h after burn. Unburned rats (Sham) serving as controls were subjected to the same procedures except that they were exposed to 24°C water. After burn injury, the animals were placed in warm incubators (30°C to 33°C) to prevent hypothermia and were allowed feed and water *ad libitum*. The rats were monitored for pain and distress and the survival rate of the animals was monitored over 48 h.

### 2.2. Parecoxib Treatment

The austere condition of the disaster site may delay evacuation time and limit the access for medics to perform intravenous administration. On the basis that an easier method of drug delivery is necessary, the treatment efficacy of early intramuscular administration of Parecoxib on burn injury was examined. The burned animals were injected intramuscularly (i.m.) with Parecoxib (0.1 mg/kg, 1.0 mg/kg, and 10 mg/kg) or saline at 20 min, 12 h, 24 h, and 36 h after burn. Unburned animals were also similarly injected i.m. 10 mg/kg Parecoxib as control.

### 2.3. Blood Analyses and Perfusion

At designated time points after burn injury, the animals were anaesthetized with Ketamine and Diazepam (75 and 10 mg/kg, resp., i.p.), the carotid artery was cannulated, and 0.3 mL of heparinized arterial blood was collected for blood gases analysis using the i-STAT Portable Clinical Analyzer (Abbott Laboratories Inc., NJ, USA). Blood parameters, lactate, blood urea nitrogen (BUN), and pH were measured. Three milliliters of blood with EDTA were also collected via the carotid artery for other analyses, with fluid replacement using heparinized saline, shortly before perfusion with saline. The collected blood was centrifuged and clean plasma was aliquoted and stored in −80°C for future assays. The animals were then perfused via transcardial perfusion with saline until the lungs, liver, and kidneys were clear of blood. The required organs were removed and soaked in 10% buffered formalin for fixation, prior to histological examination.

### 2.4. Histological Examination

The formalin-fixed tissue samples of lung, kidney, and liver, were dehydrated in ascending series of alcohol, cleared with xylene and embedded in paraffin wax. Paraffin sections of 4 *μ*m thick were then cut and dried in the oven overnight. For general morphology analysis, sections were stained in hematoxylin and eosin. Immunohistochemical stain of Myeloperoxidase (MPO) was also performed on lung sections. Polyclonal rabbit MPO antibody (Thermo Scientific, IL, USA) at 1 : 100 dilution was applied to the sections followed by antibody detection using the Envision+ anti-rabbit HRP polymer kit (DAKO UK Ltd., Cambridgeshire, UK). Sections were then visualized with 3,3′-diaminobenzidine, dehydrated, and mounted for viewing under microscope. A trained pathologist without prior knowledge of the experiment groups counted the MPO-positive polymorphonucleated cells in six random fields per slide using a 20x objective lens. The average value of MPO-positive cells was computed from at least five animals per experiment group.

### 2.5. Tissue Myeloperoxidase Activity

The tissues (lung, liver, and kidney) were separated and blotted on filter paper to remove excess fluid. Fifty milligrams of tissue samples were homogenized in 1 mL MPO buffer (50 mM potassium phosphate buffer, 0.5% hexadecyltrimethylammonium bromide, and pH 6.0) on ice. The homogenate was frozen in liquid nitrogen and thawed to room temperature four times before sonicating on ice. After sonication, the samples were centrifuged at 10,000 g at 4°C for 10 min. The supernatant (50 *μ*L) containing the solubilized MPO was incubated in the dark with 50 *μ*L of tetramethylbenzidine (Sigma, St. Louis, MO, USA) substrate for 2 min and the reaction was stopped with 50 *μ*L H_2_SO_4_. The absorbance was then read at 450 nm in room temperature with a colorimetric reader. The quantification of MPO was conducted using human MPO (number M-6908, Sigma, St. Louis, MO, USA) as standard.

### 2.6. Plasma Metabolic Substrates and Chemokine Expression

Concentrations of alanine aminotransferase (ALT), aspartate aminotransferase (AST), and creatinine in plasma samples were determined with a Cobas c111 reader (Roche, Rotkreuz, Switzerland) according to manufacturer's instructions. Plasma samples were also assayed for C-reactive protein (CRP; Biovendor, Brno, Czech Republic), interleukin-6 (IL-6; R&D Systems, MN, USA), cytokine-induced neutrophil chemoattractant-1 (CINC-1; R&D Systems, MN, USA), and prostaglandin E2 (PGE2, Cayman Chemical, MI, USA) according to manufacturers' instructions.

### 2.7. Lung Cyclooxygenase-2 and Prostaglandin E2 Expression

The actions of Parecoxib on burn-induced lung injury were examined. The lung homogenate of the animal samples was assayed for cyclooxygenase-2 (COX-2; IBL International, Hamburg, Germany) and prostaglandin E2 (PGE2, Cayman Chamical, MI, USA) using enzyme-linked immunosorbent assay technique according to manufacturer's instructions.

### 2.8. Statistical Analysis

Data are presented as mean ± standard error mean (SEM). Statistical analysis was performed by one-way analysis of variance (ANOVA, post-hoc Bonferroni test) for comparisons among multiple groups. The survival rate of the animals was compared using Kaplan-Meier analysis and statistical significance was calculated using Wilcoxon (Gehan) statistic (SPSS 15.0, IBM Corporation, NY, USA). For comparisons between 2 groups, the independent samples *t*-test was performed. *P*  value < 0.05 was considered statistically significant.

## 3. Results

### 3.1. Survival Rate

**Figure 1:**
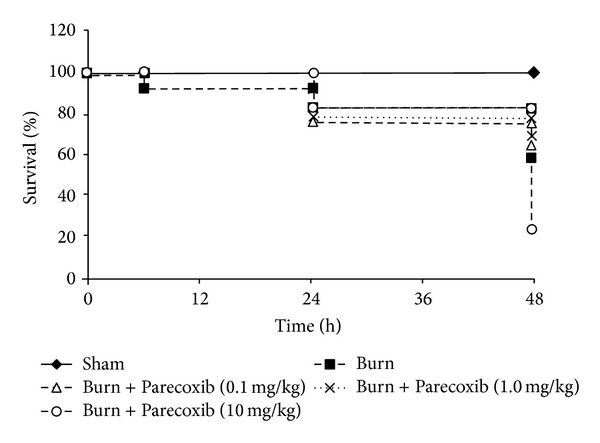
Effect of intramuscularly administered Parecoxib on survival rate of burned animals over 48 h. Animals were divided into five groups of thirty animals per group. Animals in the burn groups were exposed to 98°C to 100°C water, scalding the back for 10 s and the abdomen for 4 s. Hartmann's solution (40 mL/kg) was administered i.p. half at 1 h after burn and the remaining half at 8 h after burn. The burned animals were injected i.m. with saline, 0.1 mg/kg, 1.0 mg/kg, or 10 mg/kg Parecoxib at 20 min, 12 h, 24 h, and 36 h after burn. Sham animals were exposed to 24°C water and similarly injected i.m. 10 mg/kg Parecoxib as control. The survival rate of the animals was compared using Wilcoxon (Gehan) statistic.

The survival rate of the animals was compared with Kaplan-Meier analysis as shown in Figure 1. Burned animals had 56.5% survival after 48 hours following burns. In comparison to the control animals, the rats treated with 0.1 mg/kg and 1.0 mg/kg Parecoxib showed improved survival of 62.5% (*P*  value = 0.270) and 66.6% (*P*  value = 0.136), respectively. The surviving burn animals treated with 1.0 mg/kg Parecoxib were found to have reduced weight loss and increased food and water intake compared to the rest of the burn groups (personal observation). Burned animals treated with 10 mg/kg Parecoxib had significantly lower (*P*value = 0.001) survival rate (23.5%) compared to the burn control animals.

### 3.2. Parameters for Acute Lung Injury

**Figure 2:**
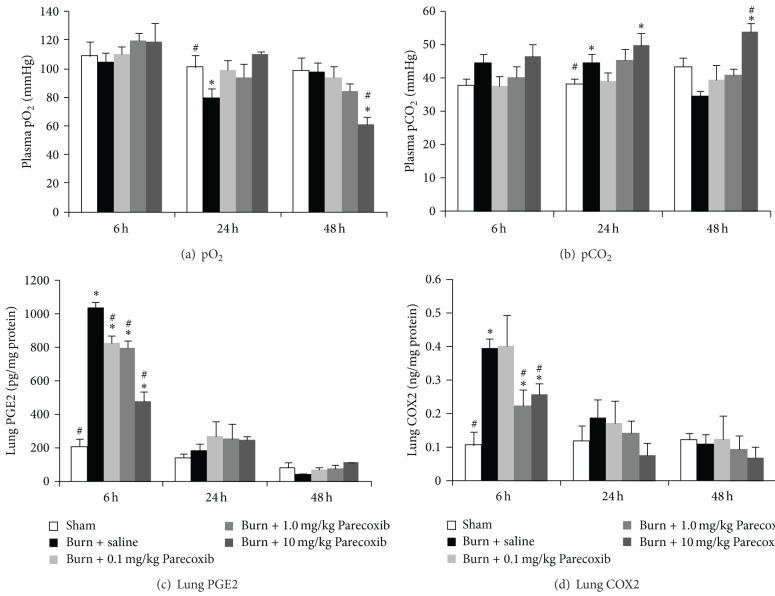
Changes in arterial blood gases and metabolic parameters over 48 h after burn injury. (a) pCO_2_; (b) pCO_2_; (c) lung PGE2; and (d) lung COX2. *Significantly different from Sham group. ^#^Significantly different from burn + saline group (*P* < 0.05, one-way ANOVA, Bonferroni post-hoc test).

Figure 2 shows analyses of blood gases, lung PGE2 levels, and lung COX2 levels of the burned animals over a period of 48 h. The acute lung injury of the burn animals was examined. It was found that 24 h following burn induced decreased pO_2_ and increased pCO_2_ in arterial blood of animals receiving saline injection. Treatment of 0.1 mg/kg and 1.0 mg/kg Parecoxib did not seem to attenuate the burn-induced changes in the blood gases. However, burn animals treated with 10 mg/kg Parecoxib showed deleterious effects of further decreased pO_2_ and elevated pCO_2_ up to 48 h compared to the burn control animals. As a COX-2 selective inhibitor, Parecoxib reduces inflammation via decreasing COX-2 activity and the downstream PGE2 formation. The lung PGE2 and COX-2 levels were significantly higher in the burn + saline group at 6 h when compared to the Sham animals and the levels gradually decreased to normal level 48 h (Figures 2(c) and 2(d)). Treatment of Parecoxib dose dependently reduced the level of PGE2 in 6 h following burn with 1.0 mg/kg Parecoxib and 10 mg/kg Parecoxib induced about 20 and 40 percent reduction, respectively.

### 3.3. Neutrophil Infiltration in the Lungs of Burned Animals

**Figure 3:**
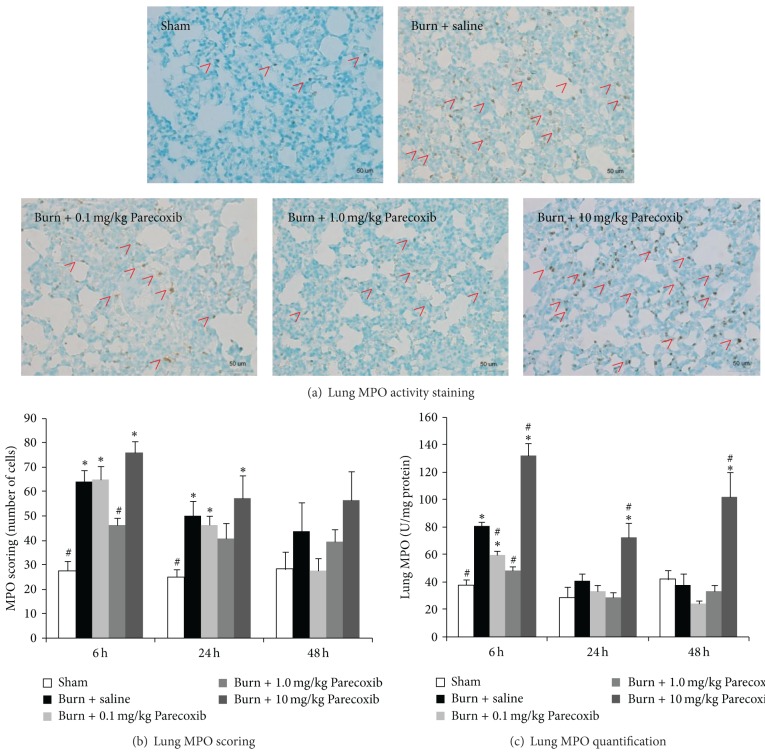
MPO quantification in lung tissue as an indicator for pulmonary neutrophil sequestration. (a) MPO activity staining at 6 h, (b) lung tissue stained for MPO and number of MPO positive cells quantified, and (c) MPO activity of the lung tissue. Red arrowhead represents neutrophil infiltration. *Significantly different from Sham group. ^#^Significantly different from burn + saline group (*P* < 0.05, one-way ANOVA, Bonferroni post-hoc test).

The MPO levels in the lungs of the burned rats were examined.  Figure 3(a) showed the representative histological MPO staining of lungs at 6 h following burn and the number of stained cell was quantified at various time points (Figure 3(b)). Burned animals treated with saline had increased levels of MPO activity in the lung at 6 h and the level gradually decreased over 48 h. The anti-inflammatory action of Parecoxib was dose-dependent with 1.0 mg/kg Parecoxib showing highest reduction in neutrophil infiltration in the burned animals and 10 mg/kg group had increased lung inflammation. The lung MPO activity of the lung samples was also quantified by colorimetric assay as shown in Figure 3(c).

### 3.4. Blood Transaminases and Liver MPO Activity

**Figure 4:**
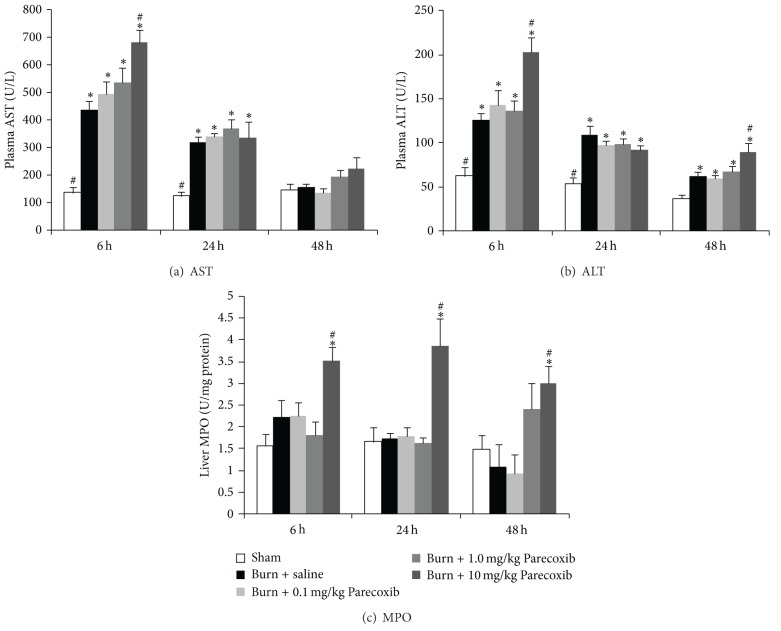
Changes in parameters for liver function over 48 h after burn injury. (a) AST; (b) ALT; (c) MPO. *Significantly different from Sham group. ^#^Significantly different from burn + saline group (*P* < 0.05, one-way ANOVA, Bonferroni post-hoc test).

The plasma AST and ALT increased significantly at 6 h after burn and gradually decreased over 48 h. At 48 h, the control burn animals showed significantly higher plasma ALT level (Figures 4(a) and 4(b)). Burn injury did not attenuate hepatic neutrophil infiltration as the liver MPO level in the burned animals remained comparable to the Sham animals over 48 h (Figure 4(c)). As expected, treatment of 0.1 mg/kg and 1.0 mg/kg Parecoxib did not seem to attenuate the burn-induced changes in the parameters for liver function at 6 h. However, burn animals treated with high dose of Parecoxib (10 mg/kg) showed deleterious effects of further increases in AST, ALT, and liver MPO at 6 h. The MPO levels remained significantly higher in the 10 mg/kg Parecoxib-treated burn animals at 24 h and 48 h. Elevated ALT level is a specific indicator for hepatic injury [17, 18]. It was reported that overdose of several drugs (including NSAIDs) is associated with elevated ALT levels and hepatotoxicity [19–22]. The data seems to suggest that high dose of Parecoxib (10 mg/kg) inflicted liver damage in burn animals in a similar manner to other NSAIDs.

### 3.5. Parameters for Acute Renal Damage

**Figure 5:**
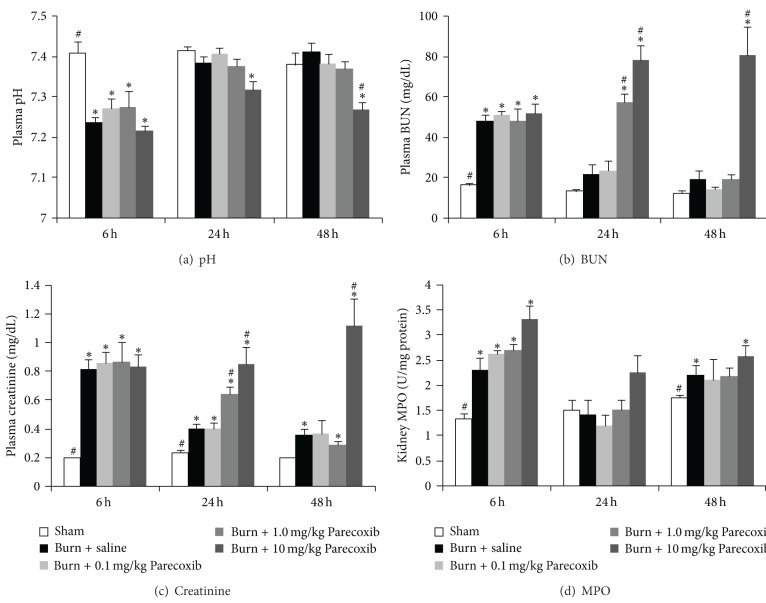
Changes in parameters for kidney function over 48 h after burn injury. (a) Plasma pH; (b) BUN; (c) plasma creatinine; and (d) kidney MPO. *Significantly different from Sham group. ^#^Significantly different from burn + saline group (*P* < 0.05, one-way ANOVA, Bonferroni post-hoc test).

The arterial plasma pH of the burn animals dropped significantly at 6 h and was restored back to Sham level 24 h after burn injury (Figure 5(a)). The plasma creatinine, BUN, and kidney MPO levels raised significantly 6 h following burn and decreased gradually over 48 h (Figures 5(b)–5(d)). Treatment of 0.1 mg/kg and 1.0 mg/kg Parecoxib did not seem to attenuate the burn-induced changes in the parameters for kidney function at 48 h. However, burn animals treated with 10 mg/kg Parecoxib showed deleterious effects of further decrease in arterial plasma pH and increases in plasma creatinine, BUN, and kidney MPO compared to the burn control animals.

### 3.6. Plasma Cytokines

**Figure 6:**
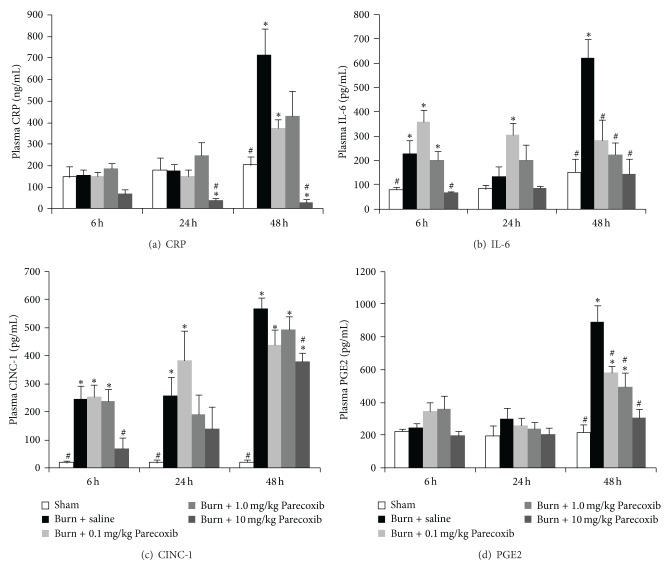
Changes in blood proinflammatory cytokines over 48 h after burn injury. (a) CRP; (b) IL-6; (c) CINC-1; and (d) PGE2. *Significantly different from Sham group. ^#^Significantly different from burn + saline group (*P* < 0.05, one-way ANOVA, Bonferroni post-hoc test).

The plasma proinflammatory cytokines profile over 48 h following burn was examined. Figures 6(a)–6(d) showed that plasma CRP, IL-6, CINC-1, and PGE2 were elevated gradually and peaked at 48 h following burn. The levels of these proinflammatory cytokines were significantly lowered in Parecoxib-treated burned animals in a dose-dependent manner, with burned animals treated 10 mg/kg Parecoxib exhibiting lowest concentration of cytokines.

### 3.7. Histological Examination

**Figure 7:**
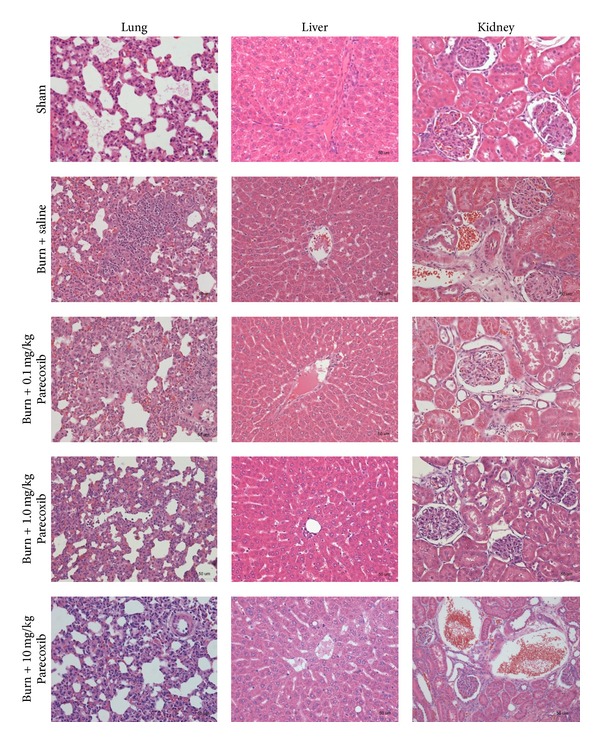
Histological examination of lung, liver, and kidney in Sham, burn + saline, burn + 0.1 mg/kg Parecoxib, burn + 1.0 mg/kg Parecoxib, and burn + 10 mg/kg, at 6 h following burn.

Histological examination revealed that the major organs such as lung, liver, and kidney in animals of Sham group appeared structurally normal (Figure 7). Compared with the organs obtained from the Sham animals, significant tissue injuries were observed in all burn animals. At 6 h following burn, the liver and kidney showed oedema with loss of normal tissue structure. Pulmonary alveolar septal thickening and infiltration of inflammatory cells were also observed. Among all Parecoxib treated groups, burn animals treated with 1.0 mg/kg Parecoxib showed the least structural damages in these organs, while burn animals treated with 10 mg/kg showed the most severe organ damages. Similar patterns were observed at 24 h and 48 h following burn (data not shown).

## 4. Discussion

In the present study, an acute burn rat model with delayed fluid resuscitation is developed in which the animals were subjected to a 45% TBSA burn and the SIRS indicators monitored for 48 h. Many rodent models of scald burn have been previously established. In the study by Gauglitz et al., Sprague Dawley rats subjected to a 60% TBSA were tracked for immunological responses and were found to exhibit marked inflammatory response [23]. Further studies by other groups showed subsequent organ damage, including lung [24], kidney and liver [25], and heart [16], suggesting SIRS arising from the burn insult. However, the burn model in most of the studies had high mortality rate and study of SIRS was not adequate. In this study, we developed the first small animal burns model that consistently produced SIRS which developed into multiple organ dysfunctions (MODS). The fluid resuscitation in this burn model is based on the Parkland formula, devised by Baxter [26], which calculates the amount of fluid required to resuscitate a burned patient based on percentage burn (4 mL/kg/percentage burn of isotonic crystalloid). However, resuscitation with large volumes of crystalloid has numerous adverse consequences, including worsening of burn oedema and intra-abdominal hypertension [27–30]. We observed more than 50% of the burned animals survived at the end of 2 days, developed SIRS and multiple organs failure in the first six hours. This model yielded consistently elevated and quantifiable parameters damages similar to Sequential Organ Failure Assessment (SOFA) scoring. The lung, liver, and kidney damages of the survived burn animals were assessed for multiple organs failure and plasma inflammatory cytokines assessed for SIRS. The burn animals developed ALI with increased levels of lung PGE2, COX2, and MPO activity at 6 h following burn, followed by a fall in arterial pO_2_ and rise in pCO_2_ at 24 h. The burn inflicted liver damage animals with increased AST and ALT levels at 6 h and gradually declined over 48 h. Acute renal damage were also observed with changes in the blood pH, BUN, creatinine levels, and kidney MPO level during the first 6 h after burn. The indicators for MODS gradually decreased as the survived burned animals were recovering over 48 h. The results showed that early intervention on burn inflicted MODS increased the animal survival in delayed fluid resuscitation. This novel model will provide a good platform for the testing of various therapeutic agents in the early treatment of SIRS and the burn related MODS in the future.

The anti-inflammatory effects of Parecoxib on ALI and MODS were examined in the present study. Similar to other burn studies, the circulating cytokines were elevated after burn injury and peaked at 24 h or 48 h [31–33]. It is proposed that systemic inflammation is initiated by the release of local inflammatory mediators in thermal injury [34]. In this study, Parecoxib was shown to suppress tissue inflammation at 6 h after severe burn and subsequently reduced SIRS at 48 h. It was found that 1.0 mg/kg Parecoxib specifically attenuated ALI 6 h after burn. In addition, as a specific COX-2 inhibitor, Parecoxib dose dependently reduced the levels of *ex vivo* pulmonary COX-2 and PGE2 at 6 h and *in vivo* proinflammatory cytokine levels at 48 h. Burn injury results in the release of proinflammatory mediators causing local and systemic inflammation and edema. Mortality and morbidity resulting from multiple end-organ failure due to systemic inflammation after severe burn have remained the primary cause of death in critically injured individuals, with the lungs being the first organ to fail [2, 3]. The most common cause of death in a burn casualty is not from direct damage to the tissue but from complications of respiratory failure. Parecoxib has been widely adopted by both the European and Australia anaesthesia and intensive care community being the first parenteral COX2 specific NSAID that can be used to alleviate postoperative pain [35, 36]. The treatment of intravenous and intramuscular of Parecoxib for acute postoperative pain in adults was reviewed and concluded from several clinical studies that the effective dose is between 0.5 to 0.8 mg/kg, corrected by weight [37]. The dose of 1.0 mg/kg Parecoxib, observed to mitigate ALI and SIRS in the burn rats, is similar to the reported efficacious dose, making it a practical anaesthesia and anti-inflammatory pharmacological agent against ALI for burn patients. The anti-inflammatory effect of Parecoxib was similarly reported in burn-injured WT mice and PPT-A^−^/^−^ mice injected with substance P [9].

However, it is also revealed that high dose Parecoxib (10 mg/kg) inflicted higher mortality compared to the control burn animals. Despite the significant decrease in levels of plasma cytokines and prostaglandin E2, high dose Parecoxib paradoxically aggravates burn-induced MODS. This finding that can be explained by prostaglandin E2 is a potent vasodilator and has autoregulatory control of blood flow to many tissues including the lungs, liver, and kidney [38]. It is well documented that prostaglandin inhibitors, NSAIDs, have minimal renal hemodynamic effect in basal condition, as prostaglandin level is low [39, 40]. In pathological conditions of water depletion, synthesis of prostaglandin is stimulated by vasoconstrictors such as angiotensin II and norepinephrine [41]. NSAID administration, in such conditions, reduces tissue perfusion and exacerbates underlying organ dysfunction [42, 43]. Furthermore, pharmacokinetic analysis of these burn animals showed that the median peak serum Parecoxib concentration was reached 15 min after injection and significant changes were observed in the bioavailability and mean residence time of Parecoxib between the burn and nonburn rats receiving 10 mg/kg Parecoxib but not in the animals which receiving 1.0 mg/kg (data not shown). The data suggest that the burn animals received high dose Parecoxib had reduced hepatic metabolism and renal clearance of the drug.

## 5. Conclusion

The pathophysiology of MODS derived from burn-induced SIRS is extensively studied and the role of mediators which control the inflammatory response is largely understood. The use of anti-inflammatory agents to suppress burn-induced SIRS has not been successful due to the complex network and multiple actions of cytokines [44–46]. At present, no specific therapeutic agent is available to attenuate SIRS to burn injury. In this study, Parecoxib is shown to mitigate systemic and lung inflammation without significantly affecting other organs in the delayed fluid resuscitation burn model. The results suggest that Parecoxib may have the potential to be used both as an analgesic and ameliorate the effects of lung injury following burn. The therapeutic use of Parecoxib for ALI in severe burn casualty is possible, but its adverse effects should be closely monitored.

## References

[1] Demling RH (2005). The burn edema process: current concepts. *Journal of Burn Care and Rehabilitation*.

[2] Turnage RH, Nwariaku F, Murphy J, Schulman C, Wright K, Yin H (2002). Mechanisms of pulmonary microvascular dysfunction during severe burn injury. *World Journal of Surgery*.

[3] Dancey DR, Hayes J, Gomez M (1999). ARDS in patients with thermal injury. *Intensive Care Medicine*.

[4] Ipaktchi K, Mattar A, Niederbichler AD (2007). Attenuating burn wound inflammation improves pulmonary function and survival in a burn-pneumonia model. *Critical Care Medicine*.

[5] Murphy TJ, Paterson HM, Kriynovich S (2005). Linking the “two-hit” response following injury to enhanced TLR4 reactivity. *Journal of Leukocyte Biology*.

[6] He L-K, Liu LH, Hahn E, Gamelli RL (2001). The expression of cyclooxygenase and the production of prostaglandin E2 in neutrophils after burn injury and infection. *Journal of Burn Care and Rehabilitation*.

[7] FitzGerald GA (2003). COX-2 and beyond: approaches to prostaglandin inhibition in human disease. *Nature Reviews Drug Discovery*.

[8] Wilgus TA, Ross MS, Parrett ML, Oberyszyn TM (2000). Topical application of a selective cyclooxygenase inhibitor suppresses UVB mediated cutaneous inflammation. *Prostaglandins and Other Lipid Mediators*.

[9] Sio SWS, Ang SF, Lu J, Moochhala S, Bhatia M (2010). Substance p upregulates cyclooxygenase-2 and prostaglandin E metabolite by activating ERK1/2 and NF-*κ*B in a mouse model of burn-induced remote Acute lung injury. *Journal of Immunology*.

[10] Ozaki-Okayama Y, Matsumura K, Ibuki T (2004). Burn injury enhances brain prostaglandin E2 production through induction of cyclooxygenase-2 and microsomal prostaglandin E synthase in cerebral vascular endothelial cells in rats. *Critical Care Medicine*.

[11] Kelsen J, Kjær K, Chen G (2006). Parecoxib is neuroprotective in spontaneously hypertensive rats after transient middle cerebral artery occlusion: a divided treatment response?. *Journal of Neuroinflammation*.

[12] Walker HL, Mason AD (1968). A standard animal burn. *Journal of Trauma*.

[13] Aikawa N, Shinozawa Y, Ishibiki K (1987). Clinical analysis of multiple organ failure in burned patients. *Burns*.

[14] Baskaran H, Yarmush ML, Berthiaume F (2000). Dynamics of tissue neutrophil sequestration after cutaneous burns in rats. *Journal of Surgical Research*.

[15] Singer AJ, Wang E, Taira BR, Steinhauff N, Rooney J, Zimmerman T (2011). Controlled mild hypothermia prolongs survival in a rat model of large scald burns. *Academic Emergency Medicine*.

[16] Zhang J-P, Ying X, Liang W-Y (2008). Apoptosis in cardiac myocytes during the early stage after severe burn. *The Journal of Trauma*.

[17] Green RM, Flamm S (2002). AGA technical review on the evaluation of liver chemistry tests. *Gastroenterology*.

[18] Pratt DS, Kaplan MM (2000). Evaluation of abnormal liver-enzyme results in asymptomatic patients. *The New England Journal of Medicine*.

[19] Watkins PB, Kaplowitz N, Slattery JT (2006). Aminotransferase elevations in healthy adults receiving 4 grams of acetaminophen daily: a randomized controlled trial. *Journal of the American Medical Association*.

[20] Athyros VG, Tziomalos K, Gossios TD (2010). Safety and efficacy of long-term statin treatment for cardiovascular events in patients with coronary heart disease and abnormal liver tests in the Greek Atorvastatin and Coronary Heart Disease Evaluation (GREACE) Study: a post-hoc analysis. *The Lancet*.

[21] Boelsterli UA, Zimmerman HJ, Kretz-Rommel A (1995). Idiosyncratic liver toxicity of nonsteroidal antiinflammatory drugs: molecular mechanisms and pathology. *Critical Reviews in Toxicology*.

[22] Bjorkman D (1998). Nonsteroidal anti-inflammatory drug-associated toxicity of the liver, lower gastrointestinal tract, and esophagus. *The American Journal of Medicine*.

[23] Gauglitz GG, Song J, Herndon DN (2008). Characterization of the inflammatory response during acute and post-acute phases after severe burn. *Shock*.

[24] Chen L-W, Hwang B, Chang W-J, Wang J-S, Chen J-S, Hsu C-M (2004). Inducible nitric oxide synthase inhibitor reverses exacerbating effects of hypertonic saline on lung injury in burn. *Shock*.

[25] Iseri SÖ, Ersoy Y, Gedik N, Ercan F, Alican I (2008). Protective role of adrenomedullin in burn-induced remote organ damage in the rat. *Regulatory Peptides*.

[26] Baxter CR (1975). Management of fluid volume and electrolyte changes in the early postburn period. *Geriatrics*.

[27] Greenhalgh DG, Warden GD (1994). The importance of intra-abdominal pressure measurements in burned children. *Journal of Trauma*.

[28] Latenser BA, Kowal-Vern A, Kimball D, Chakrin A, Dujovny N (2002). A pilot study comparing percutaneous decompression with decompressive laparotomy for acute Abdominal Compartment Syndrome in thermal injury. *Journal of Burn Care and Rehabilitation*.

[29] Hobson KG, Young KM, Ciraulo A, Palmieri TL, Greenhalgh DG (2002). Release of abdominal compartment syndrome improves survival in patients with burn injury. *Journal of Trauma: Injury, Infection and Critical Care*.

[30] Oda J, Yamashita K, Inoue T (2006). Resuscitation fluid volume and abdominal compartment syndrome in patients with major burns. *Burns*.

[31] Finnerty CC, Przkora R, Herndon DN, Jeschke MG (2009). Cytokine expression profile over time in burned mice. *Cytokine*.

[32] Kataranovski M, Magič Z, Pejnovič N (1999). Early inflammatory cytokine and acute phase protein response under the stress of thermal injury in rats. *Physiological Research*.

[33] Gauglitz GG, Song J, Herndon DN (2008). Characterization of the inflammatory response during acute and post-acute phases after severe burn. *Shock*.

[34] Rodriguez JL, Miller CG, Garner WL (1993). Correlation of the local and systemic cytokine response with clinical outcome following thermal injury. *Journal of Trauma*.

[35] Gehling MHG, Luesebrink T, Kulka PJ, Tryba M (2009). The effective duration of analgesia after intrathecal morphine in patients without additional opioid analgesia: a randomized double-blind multicentre study on orthopaedic patients. *European Journal of Anaesthesiology*.

[36] Luscombe KS, McDonnell NJ, Muchatuta NA, Paech MJ, Nathan EA (2010). A randomised comparison of parecoxib versus placebo for pain management following minor day stay gynaecological surgery. *Anaesthesia and Intensive Care*.

[37] Lloyd R, Derry S, Moore RA, McQuay HJ (2009). Intravenous or intramuscular parecoxib for acute postoperative pain in adults. *Cochrane Database of Systematic Reviews*.

[38] Piper P, Vane J (1971). The release of prostaglandins from lung and other tissues. *Annals of the New York Academy of Sciences*.

[39] Whelton A (2000). Renal and related cardiovascular effects of conventional and COX-2-specific NSAIDs and non-NSAID analgesics. *American Journal of Therapeutics*.

[40] Harris RC, McKanna JA, Akai Y, Jacobson HR, Dubois RN, Breyer MD (1994). Cyclooxygenase-2 is associated with the macula densa of rat kidney and increases with salt restriction. *Journal of Clinical Investigation*.

[41] Mori T, Cowley AW, Ito S (2006). Molecular mechanisms and therapeutic strategies of chronic renal injury: physiological role of angiotensin II-induced oxidative stress in renal medulla. *Journal of Pharmacological Sciences*.

[42] Murray MD, Brater DC (1993). Renal toxicity of the nonsteroidal anti-inflammatory drugs. *Annual Review of Pharmacology and Toxicology*.

[43] Brater DC (1999). Effects of nonsteroidal anti-inflammatory drugs on renal function: focus on cyclooxygenase -2-selective inhibition. *American Journal of Medicine*.

[44] Arturson G (1996). Pathophysiology of the burn wound and pharmacological treatment. The Rudi Hermans Lecture, 1995. *Burns*.

[45] Bhatia M, Moochhala S (2004). Role of inflammatory mediators in the pathophysiology of acute respiratory distress syndrome. *Journal of Pathology*.

[46] Dahiya P (2009). Burns as a model of SIRS. *Frontiers in Bioscience*.

